# Factors influencing referral pathways and collaboration between traditional and mental health practitioners in Sub-Saharan Africa: a scoping review

**DOI:** 10.1007/s00127-026-03173-6

**Published:** 2026-07-23

**Authors:** Tebatso Latela, Rudo Ramalisa, Fhumulani Mavis Mulaudzi

**Affiliations:** Faculty of Health Sciences, Department of Nursing, University of Pretoria, Pretoria, South Africa

**Keywords:** Traditional health practitioners, Mental health care practitioners, Collaboration, Referral pathways

## Abstract

**Purpose:**

In Sub-Saharan Africa, traditional and biomedical mental health systems coexist, yet referral pathways and collaboration between Traditional Health Practitioners and Mental Health Care Practitioners remain inconsistent. Understanding factors shaping these pathways is essential for improving continuity, cultural responsiveness, and access to mental health care. This scoping review aimed to map and synthesize evidence on factors influencing referral pathways and collaboration between Traditional Health Practitioners and Mental Health Care Practitioners in Sub-Saharan Africa.

**Methods:**

A scoping review was conducted following the Arksey and O’Malley framework, enhanced by Levac et al., and reported using PRISMA-ScR guidelines. Electronic databases, including EBSCOhost, PubMed, Scopus, and Google Scholar, were searched for English-language, peer-reviewed studies published between 2012 and 2025. Studies examining referral pathways, collaboration, barriers, or enablers between Traditional Health Practitioners and Mental Health Care Practitioners within Sub-Saharan Africa were included. Data were charted and synthesized using thematic analysis.

**Results:**

Nineteen studies met the inclusion criteria. The findings revealed barriers to collaboration and referral, including a lack of trust between practitioners, weak policy implementation, stigma, differing conceptualizations of mental illness, communication and educational gaps, resource constraints, and patient cultural preferences. Enablers included informal referral practices and practitioners’ willingness to collaborate.

**Conclusion:**

Referral pathways and collaboration between Traditional Health Practitioners and Mental Health Care Practitioners were influenced by systemic, relational, and cultural factors. While formal referral structures remain limited, informal collaboration and practitioner willingness present opportunities to strengthen continuity of care. Strategies to enhance culturally responsive, policy-supported collaboration are needed to improve mental health outcomes and reduce treatment gaps in Sub-Saharan Africa.

## Introduction and background

Mental health diseases continue to pose a substantial public health challenge and contribute substantially to disability, reduced quality of life, and increasing healthcare demands [1]. Despite advances in mental health services, many people in low- and middle-income countries continue to experience limited access to formal mental health care [1]. As a result, individuals often seek support from multiple healthcare systems, including traditional and biomedical services [2–5]. In many Sub-Saharan Africa (SS) communities, traditional and biomedical mental health systems coexist, with Traditional Health Practitioners (THPs) and Mental Health Care Practitioners (MHCPs) both playing important roles in the management of mental illness [3, 4].

THPs are defined as people recognized by the community for providing healthcare using traditional knowledge and skills rooted in African cultural beliefs [6]. They rely on spiritual, cultural, and herbal methods, viewing mental illness as linked to social or supernatural factors [7]. These practitioners may include herbalists, diviners, faith healers, and spiritual healers who often interpret mental illness through spiritual, cultural, and social perspectives [6]. In contrast, MHCPs are formally trained practitioners, such as psychiatrists, psychologists, psychiatric nurses, occupational therapists, and social workers, who assess, diagnose, and manage mental health conditions using biomedical and evidence-based approaches [8]. Although both practitioners aim to improve mental health outcomes, their differences in worldview, training, and treatment approaches may often create tension. This tension may result in negative outcomes, such as unwillingness to collaborate and a lack of mutual referrals.

In SSA, the limited and inconsistent referrals and collaboration often result in delayed diagnosis and inadequate care [9]. Where referrals and collaborations do occur, communities benefit from timely, culturally acceptable, and holistic care, which improves mental health outcomes and ensures continuity of care [10]. Despite these benefits, collaboration and referrals remain limited, contributing to the global treatment gap, with over one billion people experiencing mental disorders yet 85% receiving no treatment [1, 2]. Individuals often move between THPs and MHCPs without follow-ups or coordinated care, further highlighting the importance of understanding factors that influence referral pathways and collaboration between THPs and MHCPs in SSA [11, 12].

Literature within SSA shows that referral pathways are usually one-sided; from THPs to MHCPs due to mistrust, educational disparities, and differences in explanatory models of illness [13, 14, 15, 16]. In countries such as South Africa, the Traditional Health Practitioners Act (2007) encourages collaboration, but practical integration remains limited [6, 17]. Mapping the factors influencing referral pathways and collaboration between THPs and MHCPs in SSA is therefore important for improving continuity of care, reducing stigma, and ensuring culturally responsive mental health care.

Globally, challenges in establishing effective collaboration and referral pathways between Traditional Health Practitioners (THPs) and Mental Health Care Practitioners (MHCPs) persist across diverse health systems. In many Asian countries, mental health care remains underfunded and underprioritized despite national policies, contributing to persistent treatment gaps [18]. Reliance on THPs continues even in contexts with established biomedical services, as seen in India, where approximately 19% of the population primarily seeks traditional care, and in Australia, where trust in traditional practices continues to shape help-seeking behavior despite a well-resourced mental health system [19, 20, 21]. In China, traditional practitioners are formally integrated into the health system, with Chinese herbal medicine used by an estimated 1.5 billion people worldwide [22]. Similar barriers are also evident in high-income urban and migrant contexts; in San Francisco, limited-service availability, inadequate funding, and social stigma constrain collaboration between practitioners [23], while refugees and asylum seekers in Europe underutilize mental health services due to stigma, socioeconomic vulnerability, and limited culturally responsive care [24].

Despite growing recognition of the importance of collaboration between traditional and biomedical mental health systems, evidence regarding the factors influencing referral pathways and collaboration between THPs and MHCPs in SSA remains fragmented. Existing studies are dispersed across countries and healthcare contexts, with limited synthesis of evidence on the barriers, facilitators, and practices that influence collaboration and referral pathways. This scoping review, therefore, aims to map and synthesize evidence on the factors influencing referral pathways and collaboration between THPs and MHCPs in SSA. By identifying existing barriers, facilitators, and collaborative practices, the review seeks to advance understanding of how culturally responsive and coordinated mental health care can be strengthened within pluralistic healthcare systems.

## Methods

### Study design

This scoping review followed the methodological framework developed by Arksey and O’Malley. Further, it was enhanced by Levac, Colquhoun, and O’Brien [25, 26] and reported in accordance with the PRISMA-ScR (Preferred Reporting Items for Systematic Reviews and Meta-Analyses extension for Scoping Reviews) checklist. The steps in the framework are as follows: (1) identifying the research question, (2) identifying relevant studies, (3) study selection, (4) charting the data, and (5) collating, summarizing, and reporting the results. An additional consultation phase was not included as it is elective, and this review focused exclusively on mapping and synthesizing evidence from published literature. A scoping review was conducted because research on referral pathways and collaboration between THPs and MHCPs in Sub-Saharan Africa is inadequate and heterogeneous. This approach allows for mapping the existing evidence, identifying knowledge gaps, and clarifying key concepts.

### Identifying the research question

The scoping review addressed the following research questions: What factors influence the referral pathways and collaboration between THPs and MHCPs in SSA?

### Identifying relevant studies

An extensive literature search was conducted across multiple electronic databases, including EBSCOhost (MEDLINE, CINAHL, Health Source-Consumer Edition, Academic Complete, Africa-Wide Information, APA PsycInfo), Scopus, PubMed, and Google Scholar, from 2012 to 2025. Boolean operators were used in the search technique to integrate important terms for Population, Concept, and Context (PCC). Below are precise search strings and keywords for reproducibility and clarity. The primary researcher searched and imported all information into the EndNote reference manager, where duplicates were identified and systematically removed before screening. To ensure the inclusion of research that had undergone rigorous quality checks and peer review, grey literature was excluded from this review, which focused solely on peer-reviewed published literature.

This structured approach, guided by the Population, Concept, and Context (PCC) framework as illustrated in Table 1, ensured comprehensive coverage while maintaining focus on studies relevant to collaboration and referral between THPs and MHCPs.

### Full boolean search strategy

#### Population (THPs)

“Traditional Health Practitioners” OR THPs OR “Traditional healer” OR “Faith healer” OR “Herbalist” OR “Indigenous healer” OR “Diviner” OR “Spiritual healer” OR “Traditional medicine practitioner.”

#### Concept (MHCPs)

“Mental Health Care Practitioners” OR “mental health professional” OR “mental health care provider” OR Psychiatrist OR Psychologist OR “Psychiatric nurse” OR “Mental health practitioner” OR “Mental Health Personnel” OR “Mental Health Services” OR “Occupational therapist” OR “Social worker”.

#### Context (Referral and Collaboration)

“Referral Pathways” OR Collaboration OR “Referral system” OR “Patient referral” OR “Cooperation” OR “Integration” OR “Interprofessional Relations” OR “Partnership.”

#### Combined Search

(Population) AND (Concept) AND (Context).

**Google Scholar Search Examples**:

- “Traditional health practitioners” AND “mental health practitioners” AND “referral pathways” AND “collaboration” AND “Sub-Saharan Africa”.
- “Traditional medicine” AND “mental health care” AND “collaboration”.

### Limits Applied

- Sub-Saharan African countries.
- Publication years: 2012–2025.
- English language.

**Table 1:** Population, Concept, and Context (PCC) Framework Used in Study Selection

PCC	Description
Population	Traditional Health Practitioners (THPs) and Mental Health Care Practitioners (MHCPs)
Concept	Factors influencing referral pathways and collaboration between THPs and MHCPs
Context	Research conducted in Sub-Saharan Africa on mental health services, cultural dynamics, and health service delivery

This table summarizes the PCC framework applied to guide inclusion and exclusion during the scoping review

### Selection of the study

The inclusion and exclusion criteria used for study selection are presented in Table 2.

**Table 2:** Inclusion and exclusion criteria

Inclusion Criteria	Exclusion Criteria
Studies published between 2012 and 2025	Studies published before 2012
English-language studies	Non-English studies
Peer-reviewed full-text articles	Non-peer-reviewed articles (editorials, letters, newspaper articles)
Studies focusing on referral pathways and collaboration between THPs and MHCPs	Studies unrelated to mental health care
Studies conducted in Sub-Saharan Africa	Studies conducted outside Sub-Saharan Africa
Studies examining barriers, facilitators, or outcomes related to collaboration and referral	Studies not addressing referral pathways or collaboration between THPs and MHCPs

**Fig. 1:**
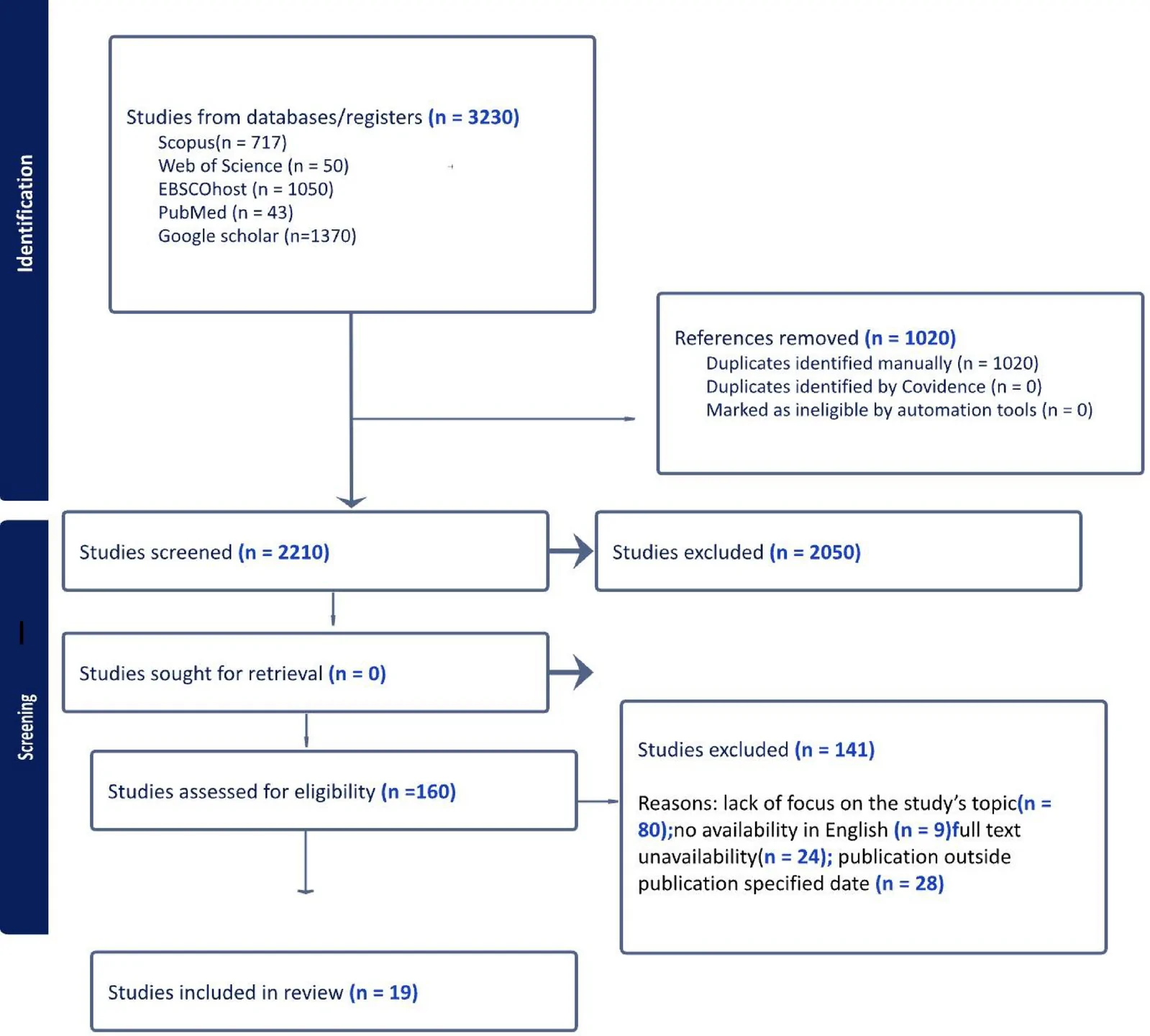
PRISMA-ScR flow diagram showing the study selection process for the scoping review. Adapted from the Covidence systematic review software

The primary researcher conducted the database searches, removed duplicates, and screened titles, abstracts, and full-text articles according to the predefined inclusion and exclusion criteria. Uncertainties regarding study eligibility were discussed with the co-authors until consensus was reached. The PRISMA-ScR framework guided the study selection process. A total of 3,230 records were identified through database searching, with no additional records retrieved from other sources (*n* = 0). After removing 1,020 duplicates, 2,210 records remained for screening. Titles and abstracts were reviewed, and 2,050 records were excluded for not meeting the inclusion criteria. The full texts of 160 articles were assessed for eligibility. Of these, 141 were excluded for reasons such as lack of focus on referral pathways or collaboration between THPs and MHCPs (*n* = 80), not being available in English (*n* = 9), full-text unavailability (*n* = 24), and publication outside the specified date range (*n* = 28). Following this process, 19 studies met the inclusion criteria and were retained for the final scoping review findings. A summary of these studies is presented in Table 3, and the study selection process is shown in Fig. 1, providing a clear visual representation of how records were identified, screened, and included.

### Charting the data

Data from the included studies were extracted and charted using a standardized data extraction framework developed by the researchers. Extracted information included author and year of publication, study title, aim, study design, population and context, data collection methods, data analysis methods, key findings, and conclusions. A summary of the included studies is presented in Table 3.

**Table 3:** Summary of studies included in the scoping review

Author(s) & Year	Title	Aim	Design	Population & Context	Data Collection	Data Analysis	Findings	Conclusion
Gureje et al., 2020	Effect of collaborative care between traditional and faith healers and primary health-care workers on psychosis outcomes in Nigeria and Ghana (COSIMPO)	To evaluate the effect of collaborative care on psychosis outcomes	Cluster RCT	THPs, faith healers, primary health care workers, patients -Nigeria & Ghana	Structured clinical assessments, interviews	Quantitative statistical analysis	Collaboration improved patient outcomes, including reduced symptom severity	Collaborative care between THPs and MHCPs is effective for psychosis management
Nyame et al., 2021	Perceptions of Ghanaian THPs, primary health care workers, service users, and caregivers regarding collaboration	To explore perceptions regarding collaboration between THPs and MHCPs	Qualitative	THPs, PHC workers, service users, caregivers-Ghana	Interviews	Thematic analysis	THPs integrate spiritual, social, and biomedical concepts in treatment	Recognizing THPs’ perspectives can inform culturally sensitive collaboration strategies
Galvin et al., 2023	Perceptions of causes and treatment of mental illness among THPs	To explore THPs’ understanding of mental illness and treatment	Qualitative	THPs-Johannesburg, South Africa	Interviews	Thematic analysis	Willingness to collaborate exists; exchange of knowledge is valued	Collaboration is supported when mutual learning and trust are prioritized
Lee et al., 2025	Perspectives of traditional healers, faith healers, and biomedical providers about mental illness treatment	To examine perspectives on mental illness treatment and potential collaboration	Qualitative exploratory study	THPs, faith healers, biomedical providers-Rural Uganda	In-depth interviews and focus group discussions	Framework analysis	Integration improved treatment adherence and symptom management	Joint care models can enhance patient outcomes
Moeta et al., 2023	Traditional health practitioners’ training needs on biomedical knowledge and skills in a South African township	To explore and describe the training needs of THPs on biomedical knowledge and skills	Qualitative exploratory descriptive study	18 THPs, City of Tshwane, Gauteng, South Africa	Lekgotla group discussion	Thematic content analysis	THPs expressed a need for biomedical knowledge, emergency care skills, improved referral abilities, and greater understanding of biomedical terminology to facilitate communication and patient care.	Training THPs in selected biomedical knowledge and skills may strengthen patient assessment, timely referral, communication, and collaboration with biomedical practitioners.
Nyembezi et al., 2024	Collaboration between THPs and biomedical health practitioners: Scoping review	To synthesize evidence on collaboration between THPs and MHCPs	Scoping review	Studies across SSA	interviews, document analysis	Thematic analysis	Barriers include hierarchy, lack of trust, and poor communication	Effective collaboration requires trust, shared understanding, and institutional support
Wollie et al., 2025	Challenges and opportunities for integrating traditional healing approaches with biomedical care for mental illness: A scoping review from healers’ perspectives	To synthesize evidence on challenges and opportunities for integrating traditional healing approaches with biomedical mental health care from traditional healers’ perspectives	Scoping review	Studies involving traditional healers and mental health care across multiple settings in SSA	Database searches of published and grey literature (2014–2024)	Thematic synthesis using NVivo	Major barriers included policy and guideline gaps, lack of recognition, educational and training challenges, differing understandings of mental illness, attitudes toward traditional healing, intellectual property concerns, and referral challenges. Enablers included healer willingness, perceived effectiveness of traditional healing, and WHO recognition of traditional medicine.	Strengthening culturally appropriate guidelines, referral systems, and collaborative frameworks may support integration between traditional healing and biomedical mental health services.
Williams et al., 2025	Pathways to mental health services across local health systems in SSA	To explore care-seeking pathways and referral systems	Systematic review	Studies across SSA	Interviews	Thematic analysis	Key barriers include differing philosophies; enablers include mutual respect and communication	Collaboration can be facilitated through structured dialogue and shared protocols
Van der Watt et al., 2017	Collaboration between biomedical and complementary and alternative care providers: barriers and pathways	To examine barriers and opportunities for collaboration	Qualitative	Biomedical providers, THPs- South Africa	Interviews	Thematic analysis	THPs and biomedical providers are willing to collaborate but need training and guidelines	Structured support can strengthen collaboration
Moshabela et al., 2016	Bridging the gap between biomedical and THPs in South Africa	To explore strategies for bridging care between THPs and biomedical providers	Qualitative	THPs and biomedical providers-South Africa	Interviews, focus groups	Thematic analysis	THPs and biomedical providers are willing to collaborate but need training and guidelines	Structured support can strengthen collaboration
Soori et al., 2024	Factors influencing integration of traditional and mainstream medicine in mental health services in West Africa	To review integration factors between traditional and mainstream medicine	Systematic review	Studies across West Africa	Literature review	Narrative synthesis	Integration influenced by policy, education, and community attitudes	Policies supporting formal referral pathways improve collaboration
Monteiro et al., 2014	Policy perspectives and attitudes towards mental health treatment in rural Senegal	To explore attitudes toward mental health care policies	Qualitative	Health providers, policymakers-Senegal	Interviews	Thematic analysis	Traditional and biomedical providers perceive policy gaps	Policy adjustments can support collaboration
Arias et al., 2016	Prayer camps and biomedical care in Ghana: Is collaboration possible?	To examine possibilities for collaboration	Qualitative case study	THPs, biomedical staff, patient-Ghana	Interviews, observations	Thematic analysis	Some THPs referred patients; biomedical staff were skeptical	Collaboration requires mutual trust and respect
Berhe et al., 2024	Traditional healing practices, factors influencing access, and the complementary effect on mental health in SSA	To review traditional healing practices and mental health outcomes	Systematic review	Studies across SSA	Literature review	Narrative synthesis	Traditional healing is widely used; access is influenced by cultural beliefs	Incorporating THPs can improve mental health service coverage
Herman et al., 2018	Closing the mental health treatment gap through collaboration in Liberia	To explore collaboration between traditional and Western medicine	Qualitative	THPs, biomedical providers-Liberia	Interviews, observations	Thematic analysis	THPs contribute unique cultural insights to care	Recognizing THPs improves culturally sensitive care
Ngubane & De Gama, 2024	Evaluation of THPs’ perspectives on mental disorders in KwaZulu-Natal	To assess knowledge, diagnosis, and treatment practices of THPs	Quantitative survey	THPs-KwaZulu-Natal, South Africa	Structured questionnaires	Statistical analysis	THPs have varied knowledge; diagnosis influenced by traditional beliefs	Training improves diagnostic accuracy and referral practices
Atewologun et al., 2025	A comprehensive review of mental health services across selected countries in sub-Saharan Africa: assessing progress, challenges, and future direction	To review mental health services, progress, challenges, and future directions in SSA	Review	Selected countries in SSA	Literature review	Narrative synthesis	Mental health services show progress but face challenges such as limited resources, workforce shortages, and gaps in policy implementation	Strategic planning, resource allocation, and integrated approaches are needed to improve mental health services and collaboration
Thobakgale et al., 2024	Traditional health practitioners’ understanding of spirit possession in Gauteng province, South Africa	To explore and interpret THPs’ understanding of spirit possession in Gauteng Province, South Africa	Hermeneutic phenomenological study	12 Traditional Health Practitioners, Gauteng Province, South Africa	In-depth individual interviews	Heidegger’s and Gadamer’s hermeneutic philosophy; Van Manen’s six-step phenomenological analysis	THPs understood spirit possession as spiritual illness, ancestral calling, demonic spirit, or witchcraft. The study highlighted differing interpretations of experiences that may be viewed as mental illness within biomedical systems. THPs also recognized the importance of referral to mental healthcare services when appropriate.	Understanding THPs’ perspectives on spirit possession may support culturally responsive mental healthcare and strengthen referral between traditional and biomedical services.
Akol et al., 2018	“We are like co-wives”: Traditional healers’ views on collaborating with the formal Child and Adolescent Mental Health System in Uganda	To explore traditional healers’ views on collaboration with biomedical health systems to improve access to child and adolescent mental health services in Uganda	Qualitative study	20 traditional healers, Uganda	Semi-structured in-depth interviews	Thematic analysis	Traditional healers expressed willingness to collaborate with biomedical practitioners but reported limited referral relationships, mutual mistrust, perceived competition, differing understandings of mental illness, and lack of policy support. Traditional healers reported referring patients to clinics, but none had received referrals from biomedical practitioners.	Collaboration between traditional healers and biomedical practitioners has potential but is hindered by mistrust, competition, and lack of formal structures to support referral and partnership.

### Collating, summarizing, and reporting the results

The extracted data were analyzed and summarized using thematic analysis. Findings from the included studies were reviewed and grouped into recurring patterns related to factors influencing referral pathways and collaboration between THPs and MHCPs. Themes and subthemes were then developed to describe the barriers and enablers influencing collaboration and referral practices across different settings in Sub-Saharan Africa.

## Results

This scoping review included 19 studies that met the inclusion criteria. A variety of methodological approaches were used in the included articles, including systematic and scoping reviews, randomized controlled trials, quantitative surveys, and qualitative studies. Most of the studies were done in South Africa (*n* = 6), Ghana (*n* = 2), and Uganda (*n* = 2). There were also studies from Senegal and Liberia, as well as regional analyses carried out throughout Sub-Saharan Africa.

Thematic analysis showed two major themes, barriers and enablers, that influence referral pathways and collaboration between MHCPs and THPs. These themes functioned at the patient, practitioner, and systemic levels. Table 4 provides an overview of the identified themes and subthemes.

Barriers to collaboration and referral included a lack of trust between practitioners, weak or inconsistent policy implementation, stigma and fear of disclosure, differing conceptualizations of mental illness, educational and communication gaps, limited resources, and patient preferences shaped by cultural beliefs. These barriers were consistently reported as limiting effective referral pathways and coordinated care across settings.

Enablers of collaboration included informal referral practices and practitioner willingness to collaborate. Several studies reported that referrals were made informally when traditional treatments were unsuccessful or when a biomedical assessment was required. Practitioner openness to collaboration supported patient movement between traditional and biomedical systems, even in the absence of formal referral structures.

**Table 4:** Themes and Subthemes from reviewed studies

Themes	Subthemes
1. Barriers to Collaboration and Referral	Trust issues between practitioners Policy and implementation challenges Stigma and fear of disclosure to MHCPs Educational, Communication, and Conceptual Gaps Resource constraints limiting collaboration Patient preferences and cultural beliefs
2. Enablers of Collaboration and Referral	Informal Referrals and Practitioner Willingness

## Discussion

### Trust Issues between practitioners

Trust emerged as an important factor influencing referral pathways and attempts toward collaboration between THPs and MHCPs. Findings from the reviewed studies showed that mistrust between practitioners was influenced by professional hierarchies, skepticism regarding the safety and effectiveness of traditional healing practices, and the historical marginalization of indigenous healing systems [27, 28, 12, 29]. Arias et al. [27] and Van der Watt et al. [29] similarly found that differences in professional perspectives and limited recognition of traditional healing practices contributed to weak engagement between traditional and biomedical practitioners. This pattern may reflect that the unequal recognition of different knowledge systems continues to influence communication between practitioners and may hinder the development of formal referral pathways within mental healthcare systems.

The findings further suggest that trust is influenced not only by interpersonal relationships among practitioners but also by structural factors within healthcare systems. In the current review, the absence of formal referral structures, collaborative guidelines, and shared communication platforms appeared to reinforce fragmented care and inconsistent referral practices. Therefore, referrals between THPs and MHCPs were often reported as informal, inconsistent, or limited across several studies included in the review [29].

Despite these challenges, Nyame et al. [12] and Herman et al. [28] reported that some practitioners expressed openness toward future collaboration when mutual respect and recognition of different knowledge systems were encouraged. Although these findings do not suggest the existence of well-established collaborative systems, they may indicate opportunities to strengthen communication and develop more structured referral pathways in the future. Evidence from collaborative initiatives in other healthcare contexts, including HIV and maternal healthcare services, further suggests that co-training and shared case management may support trust-building across practitioner groups [30, 31]. Collectively, these findings emphasize the importance of culturally respectful and inclusive approaches when considering future efforts to strengthen referral pathways and continuity of mental healthcare within pluralistic healthcare systems.

### Policy and implementation challenges

The findings of this review suggest a persistent gap between policy recognition and the practical implementation of referral pathways and collaboration between THPs and MHCPs in SSA. Although countries such as South Africa, Ghana, and Nigeria formally recognize traditional practitioners within national health frameworks, clear operational guidelines for referral and collaboration remain limited [32]. Similar findings were reported by Van der Watt et al. [29] and Soori et al. [33], who identified weak institutional support and limited formal structures for collaboration between traditional and biomedical practitioners. This pattern may show that policy recognition alone may be insufficient to support structured referral pathways within mental healthcare systems.

In the current review, the absence of clear referral procedures and collaborative frameworks appeared to contribute to informal and inconsistent referral practices between THPs and MHCPs. As a result, practitioners often appeared to rely on personal judgment and informal communication when navigating patient referrals [12, 27]. This finding is consistent with evidence from Senegal, where the existence of a national mental health plan was accompanied by challenges in coordinated implementation and service delivery, highlighting the difficulties of translating policy intentions into routine practice [34]. This may contribute to fragmented mental healthcare pathways and inconsistent continuity of care across different settings.

Despite these challenges, the formal recognition of THPs within policy frameworks may provide a foundation for future efforts to strengthen referral pathways and collaboration. However, the findings of this review suggest that future efforts may require practical and contextually appropriate referral guidelines and clearer communication structures to support more coordinated mental healthcare practices within pluralistic healthcare systems in SSA. The absence of operational referral structures may have further reinforced the informal and inconsistent referral practices identified across several themes in this review.

### Stigma and fear of disclosure

Stigma emerged as an important factor influencing referral pathways and engagement between THPs and MHCPs. Findings from Nyame et al. [12], Arias et al. [27], and Herman et al. [28] showed that some patients hesitated to disclose prior consultations with THPs to MHCPs due to fear of judgment or being perceived negatively within biomedical healthcare settings. These findings suggest that concerns regarding disclosure may influence communication between patients and practitioners and may contribute to incomplete clinical histories and disruptions in continuity of care. The dismissal of traditional healing practices as unscientific within some biomedical contexts also appeared to reinforce patient reluctance to discuss the use of traditional mental healthcare services [12, 27].

The findings further suggest that stigma may operate at multiple levels, including patient, practitioner, and healthcare system levels. Wollie et al. [35] similarly reported that negative perceptions surrounding traditional healing practices may affect engagement between traditional and biomedical healthcare systems. In the current review, stigma appeared to influence not only patient disclosure but also broader interactions between THPs and MHCPs, particularly in contexts where traditional healing practices were not formally recognized or respected within mental healthcare settings.

Collectively, these findings highlight how stigma and fear of disclosure may continue to influence referral pathways and attempts toward coordinated mental healthcare within pluralistic healthcare systems in SSA.

### Educational, communication, and conceptual gaps

Differences in training and explanatory models of mental illness emerged as important factors influencing referral pathways and engagement between THPs and MHCPs. Arias et al. [27], Nyame et al. [12], and Van der Watt et al. [29] reported that MHCPs generally approached mental illness from biomedical perspectives. In contrast, THPs often interpreted mental illness through spiritual, cultural, and social lenses [36]. These differing understandings of mental illness appeared to contribute to parallel rather than integrated approaches to mental healthcare. The findings further suggest that differences in professional language, terminology, and documentation practices may have limited communication between practitioners and contributed to inconsistent referral practices.

The findings of this review also suggest that educational and communication gaps may reflect broader differences in how traditional and biomedical knowledge systems are recognized within mental healthcare settings. Van der Watt et al. [29] similarly reported that differing professional perspectives and limited mutual understanding affected practitioners’ engagement. In the current review, the limited opportunities for shared communication and learning appeared to reinforce separation between traditional and biomedical mental healthcare approaches.

Although the reviewed studies provided limited evidence of formal educational initiatives to improve collaboration, the findings may indicate the importance of creating opportunities for dialogue and mutual understanding among practitioners across different healthcare systems [10, 9]. Collectively, these findings highlight how educational, communication, and conceptual differences may continue to influence referral pathways and attempts toward more coordinated mental healthcare practices within pluralistic healthcare systems in SSA.

These findings may also help explain the mistrust and inconsistent referral practices reported across the reviewed studies, as differing understandings of mental illness appeared to limit mutual recognition and communication between practitioners.

### Resource constraints

Limited funding, infrastructure, and workforce capacity emerged as factors influencing referral pathways and engagement between THPs and MHCPs across settings [33]. Similar resource-related challenges were reported in Senegal, where limited personnel, inadequate mental health resources, and funding constraints were identified as barriers to strengthening mental healthcare services and implementation efforts [33, 37]. These constraints appeared to reduce opportunities for joint training, formal referral mechanisms, and sustained communication between practitioners. The findings of this review therefore suggest that resource limitations may continue to affect efforts toward developing structured referral pathways within mental healthcare systems in SSA.

However, evidence suggests that collaboration and referrals are not solely determined by resource availability. Informal networks and practitioner initiative have enabled referral despite resource limitations [32, 27]. This may suggest that referral practices between THPs and MHCPs often occur informally in settings where formal referral systems remain limited.

### Patient preferences and cultural beliefs

Patient preferences and cultural beliefs emerged as important factors influencing referral pathways between THPs and MHCPs. Nyame et al. [12], Berhe et al. [5], and Galvin et al. [38] reported that many individuals initially sought care from THPs due to perceived cultural safety, trust, and holistic forms of support. These findings suggest that traditional healing practices continue to influence mental healthcare-seeking behaviors across several SSA contexts.

Nyame et al. [12], Herman et al. [28], and Thobakgale [36] further reported that some patients sought care from both THPs and biomedical practitioners depending on their beliefs, experiences, and perceptions of treatment effectiveness. Similar findings were reported by Williams et al. [39], who found that patients frequently accessed both traditional and biomedical services during their mental healthcare journeys, with help-seeking pathways influenced by cultural beliefs and perceptions of care. This may suggest that healthcare-seeking pathways within SSA settings are not fixed but shaped by cultural beliefs, accessibility, and prior experiences with care. The findings, therefore, indicate that patient movement between traditional and biomedical systems may continue to influence referral pathways within pluralistic mental healthcare contexts.

Collectively, these findings highlight the important role of cultural beliefs and patient preferences in shaping engagement with both traditional and biomedical mental healthcare systems in SSA.

### Enablers of collaboration and referral

Informal referral practices and practitioner willingness emerged as key enablers of collaboration. Soori et al. [33], Lee et al. [40], and Herman et al. [28] reported that referrals occasionally occurred when traditional treatments were perceived as unsuccessful or when additional biomedical intervention was considered necessary. Some MHCPs also recognized the importance of engaging THPs due to patients’ continued reliance on traditional forms of care [12, 28, 40, 10]. Similarly, some studies reported that traditional healers expressed willingness to collaborate with biomedical practitioners and described referral practices occurring despite the absence of formal collaborative structures [41, 42]. These findings suggest that interactions between practitioners were more likely to occur through informal and relationship-based processes rather than through formalized referral systems.

The findings further suggest that practitioners’ willingness may serve as a foundation for future efforts to strengthen referral pathways between traditional and biomedical mental healthcare systems. However, the reviewed studies provided limited evidence of structured or sustained collaborative systems across SSA settings. An exception was the COSIMPO trial conducted in Nigeria and Ghana, which demonstrated that collaboration among traditional and faith healers and primary healthcare workers was associated with improved psychosis outcomes, suggesting that more structured collaborative approaches may be feasible in some SSA contexts [33]. This may indicate that existing interactions between practitioners remain largely informal and context-dependent.

Collectively, these findings highlight that informal engagement and practitioner willingness continue to influence referral practices within pluralistic mental healthcare systems in SSA, despite the limited presence of formal referral structures.

### Strengths and limitations

This scoping review has several limitations that should be considered when interpreting the findings. First, the review was restricted to English-language, peer-reviewed studies published between 2012 and 2025. As a result, relevant evidence from earlier periods, non-English publications, and grey literature may have been excluded, potentially limiting the breadth of perspectives captured. Second, the reliance on electronic databases may have overlooked locally produced or unpublished studies, particularly those documenting informal or community-based collaboration practices. Third, studies outside the mental health field were excluded, potentially omitting insights from related health sectors where traditional and biomedical collaboration is more established. Finally, this review focused on mapping reported factors influencing referral pathways and collaboration. It did not assess how effectively these practices were implemented in real-world settings or whether they resulted in sustained improvements in mental health outcomes. These limitations reflect common challenges within this body of literature and should be borne in mind when considering the implications of the findings.

Despite these limitations, this scoping review provides a comprehensive synthesis of recent literature from 2012 to 2025 on factors influencing referral pathways and collaboration between THPs and MHCPs in SSA. Including multiple databases using the PCC framework, it maximizes coverage and identifies important and relevant themes across diverse contexts. The review also offers practical insights for improving collaboration, guiding policy, and informing nursing practice, education, and research.

## Conclusion

This scoping review highlights multiple factors influencing collaboration and referral pathways between THPs and MHCPs in SSA. Barriers such as trust issues, policy gaps, stigma, conceptual differences, communication challenges, resource constraints, patient preferences, and cultural beliefs hinder the development of effective collaboration and referral pathways. At the same time, enablers, including informal referral practices and practitioners’ willingness, demonstrate that collaboration and referrals are possible even in contexts with limited institutional and systemic support. The findings of this scoping review emphasize the importance of strategies that address not only structural and policy factors but also cultural, relational, and interpersonal dynamics. Future research should explore interventions that strengthen trust, mutual understanding, and integrated referral systems, as well as the role of nurses in facilitating collaboration through education, practice, and research initiatives.

## Data Availability

Online access to the data from the studies that were part of our scoping review is open to the public.
